# Supplementary material for: Exploring the Effects of VgPIP1;2 Overexpression in the Roots of Young Rice Plants: Modifications in Root Architecture, Transcriptomic and Metabolomic Profiles
Source: Plants (Basel). 2025 Nov 28;14(23):3628. doi: 10.3390/plants14233628 (PMC12694057; doi:10.3390/plants14233628)
Supplement: Supplementary file 1 [file plants-14-03628-s001.zip › Supplementary Table 1.pdf]

Supplementary Table 1. Primers used in this study.

Gene name	Forward Primer 5’ → 3’	Reverse Primer 5’ → 3’
VgPIP1;2	ATGGAAGGCAAGGAGGAG	TTAAGCCCTGCTTTTGAATGGAAT
M13 primer	GTAAAACGACGGCCAG	CAGGAAACAGCTATGAC
OsUBQ	ACCACTTCGACCGCCACTACT	ACGCCTAAGCCTGCTGGTT
