# Supplementary figures and images for: Exploring the Effects of VgPIP1;2 Overexpression in the Roots of Young Rice Plants: Modifications in Root Architecture, Transcriptomic and Metabolomic Profiles
Source: Plants (Basel). 2025 Nov 28;14(23):3628. doi: 10.3390/plants14233628 (PMC12694057; doi:10.3390/plants14233628)

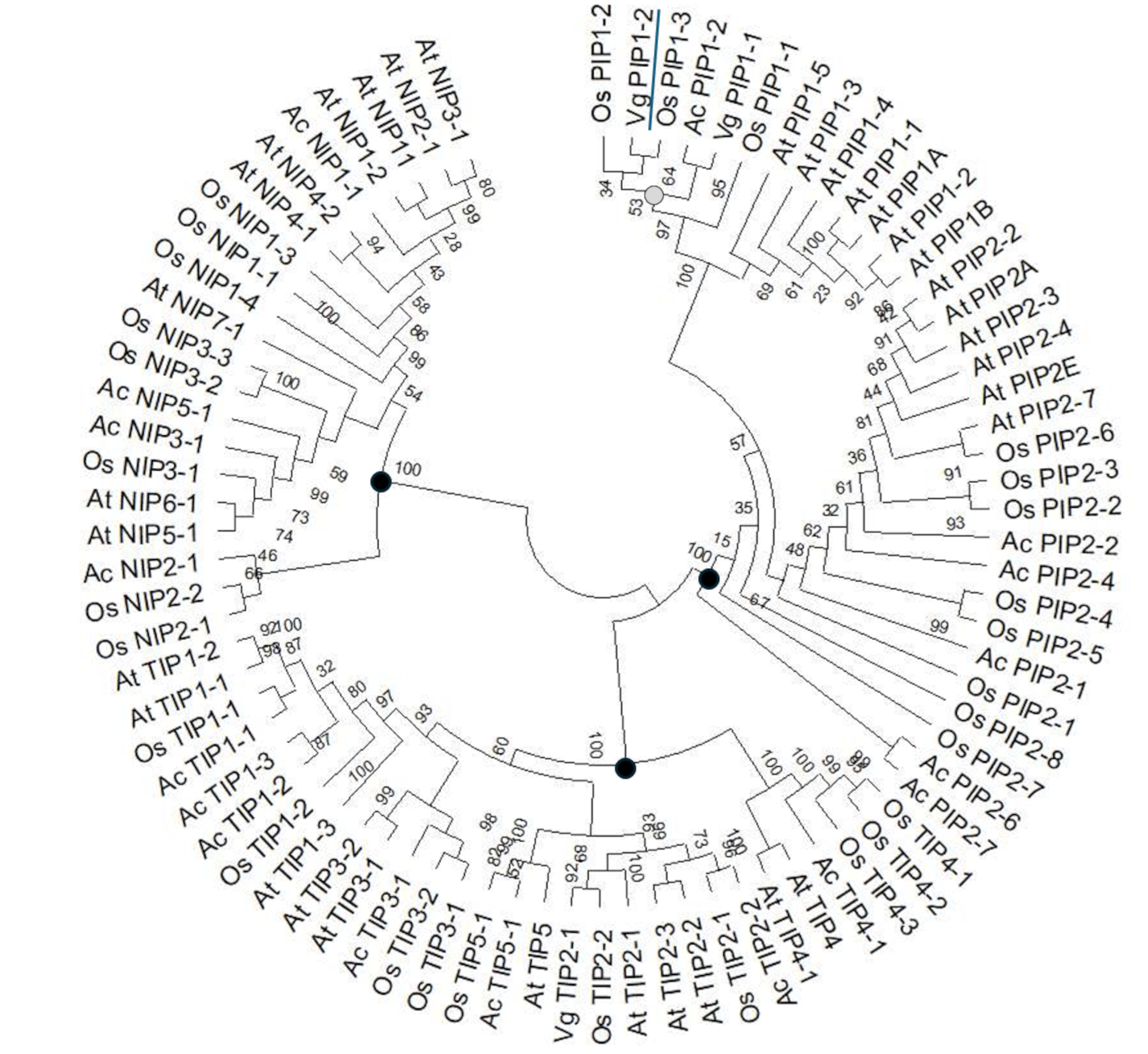

Supplement: Supplementary file 1 [file plants-14-03628-s001.zip › Supplementary Fig 1.tif]

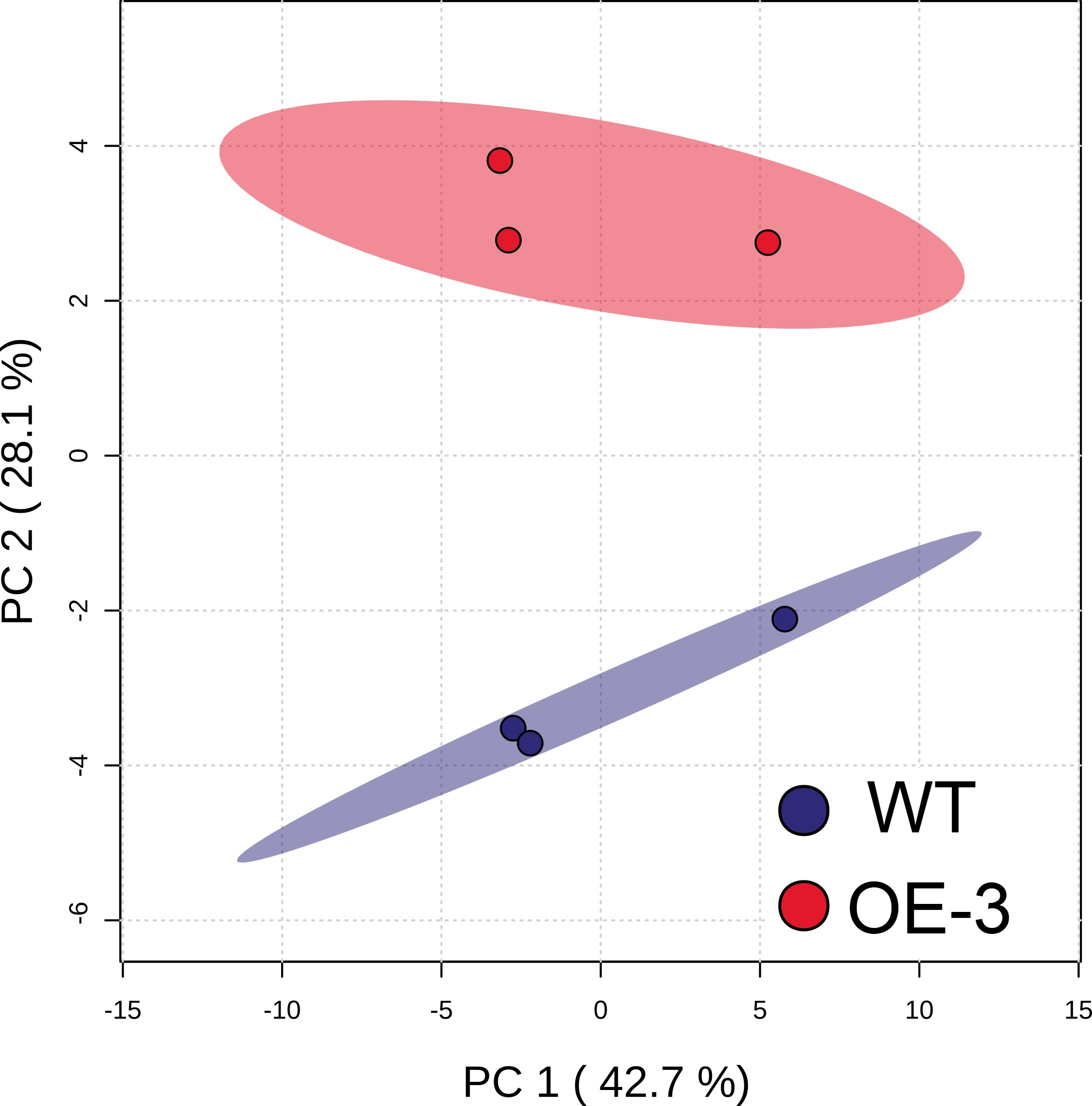

Supplement: Supplementary file 1 [file plants-14-03628-s001.zip › Supplementary Fig 2.jpg]
